# Editorial: The integrity of randomized clinical trials: consensus statements from Hong Kong to Cairo

**DOI:** 10.3389/frma.2025.1588882

**Published:** 2025-06-10

**Authors:** Khalid S. Khan, Aurora Bueno-Cavanillas, Javier Zamora

**Affiliations:** ^1^Consortium for Biomedical Research in Epidemiology and Public Health (CIBERESP), Madrid, Spain; ^2^Department of Preventive Medicine and Public Health, University of Granada, Granada, Spain; ^3^Instituto de Investigación Biosanitaria Ibs-Granada, Granada, Spain; ^4^Department of Biostatistics, Ramón y Cajal University Hospital (IRYCIS), Madrid, Spain

**Keywords:** research integrity, clinical trials, retraction, research misconduct, Cairo consensus

Research integrity requires adherence to ethical and professional principles and standards (Steneck, 2006). In 2019, the World Conference on Research Integrity endorsed the Hong Kong Principles for researchers and institutions (Moher et al., 2020). These generic integrity principles and related standards ought to be applied in biomedicine and life sciences, which underpin health research. They should be at the heart of the evidence generated to form the foundation for the practice of evidence-based medicine (EBM). Randomized clinical trials (RCT) are ranked at the top of the evidence hierarchy, but the rising numbers of RCT retractions and expressions of concern about their conduct have raised concerns that defective evidence has permeated into EBM (Khan, 2024). The RCT study design minimizes the risk of selection bias by randomly assigning participants into experimental or control groups and following them up to compare their outcomes. It follows that the integrity of RCT design, conduct, analysis and publication is critical to the trustworthiness of the evidence for EBM. There is now a recognized need for discipline specification of the generic integrity principles. The trialists involved in RCT design and conduct need robust, specific research integrity policies and guidelines. This Frontiers' Research Topic “*The Integrity of Randomized Clinical Trials*” examines integrity-related issues at various stages covering the RCT research life cycle.

Globally, there are about 48,000 English- and non-English-language journals published (around 30%, i.e., 16,000, in biomedicine), growing in the region of 3% annually (Bhosale, 2021). In peer review, much of the criticism targets biomedical and clinical research. Around 25,000–30,000 RCTs are published annually in the journals indexed in the PubMed database alone (Khan, 2024). The existence of trials with integrity flaws creates pollution in the biomedical research and life sciences ecosystem that has downstream consequences for clinical guidelines issued by professional bodies and approvals and marketing authorization given by drug and device regulators. EBM is hampered by the fact that not all published RCTs demonstrate equal rigor. There is a need to focus on RCTs because they contribute directly to healthcare practice and policy recommendations. Flawed RCTs continue to be cited and used in evidence synthesis, and corrections of systematic reviews and practice guidelines following RCT retractions are uncommon (Kataoka et al., 2022).

The articles published in the Research Topic “*The Integrity of Randomized Clinical Trials*” cover a spread across the RCT research life cycle. For example, in RCT registration, a reanalysis of trial registrations in the Chinese Clinical Trial Registry highlighted issues related to quality (Li et al.), a global problem that is not limited geographically; in RCT conduct, the challenges faced in responsible conduct require role delineation, professional development, and supportive institutional environments (Peralta and Sánchez-Santiago); in RCT publication, an analysis of trials related to traumatic brain injury in the ClinicalTrials.gov registry has raised concerns about underreporting (Guo et al.); and, in the post publication stage, the extent of retracted citations in the literature on medically assisted reproduction has been found to be low, with plagiarism being the most common flaw and RCT the most common publication type retracted in the field (Minetto et al.). Examining issues across research life cycle matters as peer review and journal editorial assessments cannot be expected to weed out defective RCTs that harbor integrity flaws in their designs and conduct. Action is needed throughout the RCT research life cycle, engaging all relevant stakeholders to align on a shared vision for promoting responsible research conduct (Butt et al., 2024).

Integrity screening of papers during peer review and in evidence syntheses has limitations due to incomplete validation of RCT integrity checklists (Khan et al., 2023; Núñez-Núñez et al., 2023). It is important to recognize that research integrity is a multi-dimensional concept, not limited to performing checks of manuscripts of completed RCTs. It spans the entire research process, including RCT conception and design, research ethics committee approval and consent, RCT conduct and analysis in compliance with the approved protocol and the registered statistical analysis plan, as well as reporting of results and correction of the published record. Integrity should be guaranteed at each one of these steps, and this is the responsibility of researchers, academic institutions, funding agencies, and publishers, among other stakeholder organizations. For this, they need RCT-specific integrity guidelines.

The generic integrity guidance contained in the 2019 Hong Kong document (Moher et al., 2020) and its subsequent versions, has limitations when it comes to RCTs (Butt et al., 2024). The research integrity recommendations contained in such documents are meant to promote responsible research conduct across scientific disciplines. However, as research culture is discipline-specific, they don't always gain acceptance due to their generic nature. For example, in Australian universities active in health and medical research according to a 2023 paper, the official codes of conduct for underpinning responsible research practice have not properly emphasized registering protocols, providing analysis codes, and discouraging p-hacking, among other transparency and openness principles (Ong et al., 2023). In this background, an international team of experts covering the entire stakeholder spectrum set out to develop the Cairo consensus statements specifically on RCT integrity. The stakeholders covered geographically all the inhabited continents of the world and both developed and under-developed settings, as well as organizations such as trialists' institutions, consumer representatives, professional bodies, funders, and journals. The Cairo integrity statements provide authoritative literature-underpinned consensus-based best research practice guidelines for the key stages of the entire RCT life cycle (Khan and Cairo Consensus Group on Research Integrity, 2023; Khan et al., 2025). The consensus will need to be an ongoing effort requiring revision and update as new evidence emerges and the RCT research discipline progresses. The recent update of the Helsinki Declaration by the World Medical Association incorporating new clauses on research integrity is a sign of travel in the right direction (World Medical Association, 2025).

It is hoped that “*The Integrity of Randomized Clinical Trials*” Research Topic, which draws attention to the need to focus on the disciplinary specification of responsible research conduct, will represent progress toward the goal of undertaking useful research for promoting EBM. It will also have a wider impact on inculcating a research integrity culture across biomedicine and life sciences. In addition to the benefits for EBM and health research, upholding scientific integrity is a necessary investment to regain and maintain the trustworthiness of scientists as a respected professional group valued by society.
